# Polish Varieties of Industrial Hemp and Their Utilisation in the Efficient Production of Lignocellulosic Ethanol

**DOI:** 10.3390/molecules26216467

**Published:** 2021-10-26

**Authors:** Aleksandra Wawro, Jolanta Batog, Weronika Gieparda

**Affiliations:** Institute of Natural Fibres and Medicinal Plants, National Research Institute, Wojska Polskiego 71B, 60-630 Poznan, Poland; jolanta.batog@iwnirz.pl (J.B.); weronika.gieparda@iwnirz.pl (W.G.)

**Keywords:** hemp biomass, alkaline pretreatment, SEM, FTIR, response surface methodology, SHF, SSF, bioethanol

## Abstract

Nowadays, more and more attention is paid to the development and the intensification of the use of renewable energy sources. Hemp might be an alternative plant for bioenergy production. In this paper, four varieties of Polish industrial hemp (Białobrzeskie, Tygra, Henola, and Rajan) were investigated in order to determine which of them are the most advantageous raw materials for the effective production of bioethanol. At the beginning, physical and chemical pretreatment of hemp biomass was carried out. It was found that the most effective is the alkaline treatment with 2% NaOH, and the biomasses of the two varieties were selected for next stages of research: Tygra and Rajan. Hemp biomass before and after pretreatment was analyzed by FTIR and SEM, which confirmed the effectiveness of the pretreatment. Next, an enzymatic hydrolysis process was carried out on the previously selected parameters using the response surface methodology. Subsequently, the two approaches were analyzed: separated hydrolysis and fermentation (SHF) and a simultaneous saccharification and fermentation (SSF) process. For Tygra biomass in the SHF process, the ethanol concentration was 10.5 g∙L^−1^ (3.04 m^3^·ha^−1^), and for Rajan biomass at the SSF process, the ethanol concentration was 7.5 g∙L^−1^ (2.23 m^3^·ha^−1^). In conclusion, the biomass of Polish varieties of hemp, i.e., Tygra and Rajan, was found to be an interesting and promising raw material for bioethanol production.

## 1. Introduction

The European Union countries have been obliged to achieve a certain share of biofuels in transport and to take measures to reduce greenhouse gas emissions. It is, therefore, necessary to replace diesel and gasoline with biofuels which are produced from lignocellulosic raw materials and represent an advantageous option for the fuels currently in use due to their renewable nature and the emission of an acceptable quality exhaust gases. Currently, mainly three biofuels are produced: bioethanol, biodiesel, and biogas. According to the EU RED II directive, the contributions of advanced biofuels and biogas produced from raw materials listed in Annex IX, part A to this directive, including lignocellulosic feedstocks as a share of final energy consumption in the transport sector are expected to be at least: 0.2% in 2022, 1% in 2025, and 3.5% in 2030 [1]. The production of biofuels from plant biomass is innovative and contributes to the solution of the key issue in the production of biofuels for transport fuels.

In Poland, high expectations are associated with plant biomass due to a significant amount of waste, including that from agri-food sector and the available acreage of agricultural land that can be used for the cultivation of energy crops. In recent years, there has been an increase in the acreage of cultivated industrial hemp (*Cannabis sativa* L.) in Poland (over 1000 ha). The cultivation of hemp for seed purposes is intensively developed, and there is unused hemp biomass in the field, which can be a suitable raw material for the production of lignocellulosic ethanol. Hemp is an environmentally friendly plant characterized by a short vegetation period (3–4 months), a rapid growth up to 4 m in height, and a dry matter yield up to 15 Mg·ha^−1^. These plants improve soil quality and are useful for the remediation of degraded land (e.g., in the region of lignite mine). Hemp is also extremely resistant, perfectly adapts to various climatic conditions, is resistant to various pests, requires a slight number of pesticide treatments, and the cultivation of 1 ha of hemp in one season absorbs approximately 11 Mg of CO_2_ from the atmosphere [2,3,4].

The dry matter yields of these hemp varieties described in this study were as follows: Białobrzeskie 8–10 Mg·ha^−1^, Tygra 8–11 Mg·ha^−1^, Henola 7–8 Mg·ha^−1^, and Rajan 9–12 Mg·ha^−1^. Based on the data from 2016 (the own research of the Institute of Natural Fibres and Medicinal Plants—National Research Institute), it was estimated that, in Poland, the acreage of devastated and degraded land requiring reclamation and constituting a potential area for hemp cultivation for energy purposes amounts to approximately 65,000 ha. In the INF&MP-NRI, the Hemp Program is carried out, the aim of which is to develop hemp cultivation for seed reproduction. As part of this program, cultivation acreage is significantly increasing every year: 420 ha in 2018, 1000 ha in 2019, and 2000 ha in 2020.

The use of hemp waste straw for the production of lignocellulosic ethanol is beneficial for the environment, as it results in a rational management of bio-waste. The straw remaining after the ginning of the hemp panicles is not suitable for textile purposes, but it can be used, e.g., as a raw material for the production of bioethanol. Additionally, the dynamic growth of the hemp cultivation acreage in Poland may significantly contribute to increasing the efficiency process of obtaining bioethanol.

The process of plant biomass conversion to lignocellulosic ethanol includes several stages, from the preparation of plant material (effective pretreatment), through enzymatic hydrolysis, i.e., the decomposition of polysaccharides into fermentable sugars (the selection of effective enzymatic preparations) and to ethanol fermentation (the selection of appropriate microorganisms). The production of bioethanol from lignocellulosic raw material consists of the deconstruction of cell walls into individual polymers and the hydrolysis of carbohydrates into simple sugars. Currently, one of the main challenges is to increase the efficiency of the fermentation of organic substrates, and alternative solutions that directly affect the quantity and the quality composition of the final product are still being researched.

Hemp biomass as a lignocellulosic raw material contains a polymer complex, i.e., lignocellulose, which is relatively resistant to biodegradation. It is found in cell walls and consists of cellulose, hemicelluloses, and lignin. Cellulose and hemicelluloses are potential substrates in the fermentation process, while lignin adversely affects the conversion of hemp biomass. This necessitates the use of the pretreatment of biomass, the purpose of which is to fragmentate the solid phase and loosen the compact structure of lignocellulose. Recent advances in biomass pretreatment can be found in the review article by Bing et al. [5]. The second important stage in the process of obtaining bioethanol from hemp biomass is enzymatic hydrolysis, which determines the amount of simple sugars metabolized by yeast in the fermentation process. The hydrolysis process can be carried out as the SHF process—separate hydrolysis and fermentation (enzymes operate at 50–60 °C)—or the SSF (process of simultaneous saccharification and fermentation), where enzymes must be adapted to the conditions of the fermentation process, i.e., 30–40 °C. The last stage in the process of hemp biomass conversion is the ethanol fermentation of the obtained hydrolysates. A method that combines cellulose hydrolysis with sugar fermentation in one bioreactor seems to be more effective and economical [6,7,8,9,10,11].

The aim of the presented study was to indicate which of the four Polish varieties of industrial hemp (Białobrzeskie, Tygra, Henola, and Rajan) are the most suitable raw materials for the effective production of lignocellulosic ethanol. Thus far, no literature has been published about the possibility of using these Polish varieties of hemp as a raw material in the process of obtaining bioethanol.

## 2. Results and Discussion

### 2.1. Hemp Biomass Preparation

The hemp biomass of the four varieties (Białobrzeskie, Tygra, Henola, and Rajan) was first cut into fragments up to 1 cm in size and then was comminuted by the knife mill for mesh sizes of 2 and 4 mm. In order to choose the most favorable fractions, the content of reducing sugars released during an enzymatic test was determined. It was found that the highest values of reducing sugars were obtained for fractions up to 2 mm, and two varieties, Tygra and Białobrzeskie, showed higher sugar values for the tested fractions compared to Rajan and Henola (Table 1).

### 2.2. Alkaline Pretreatment

The purpose of chemical pretreatment is removing lignin from materials with lignocelullose and increasing the accessibility of biomass structure. The type of a reagent used has a significant effect on the performance of the chemical pretreatment. Sodium hydroxide is one of the most popular alkaline reagents used in this process.

The optimization of the concentration of sodium hydroxide used in alkaline treatment for the four varieties of hemp biomass was carried out based on the amount of reducing sugars released after the enzymatic test. Concentrations ranging from 1.5% to 3% were tested (Table 2). It was found that, for Tygra and Białobrzeskie varieties, for 2% NaOH, the amount of released reducing sugars was about 13% higher than for 1.5%. In turn, for 3% sodium hydroxide, the content of reducing sugars was at a similar level. For Henola and Rajan varieties, a completely different correlation was observed; the lowest level of released reducing sugars was noted for the concentration of 3% NaOH, while, at the concentration of 2%, the content of reducing sugars was the highest. Moreover, it was noted that two of the varieties, i.e., Tygra and Rajan, were characterized by over 10% higher content of reducing sugars than Białobrzeskie and Henola, which proves that these are the varieties more susceptible to the alkaline pretreatment. Based on the obtained results, the concentration of sodium hydroxide at the level of 2% was selected for further research. Kumar et al. [12] conducted similar research and stated that the sodium hydroxide pretreatment of lignocellulosic biomass resulted in the highest level of delignification at 2% NaOH. In turn, Zhao et al. [10], in the research on the use of American industrial hemp for the production of bioethanol, used the alkaline treatment of 1% NaOH.

Efficient pretreatment should decrystallize cellulose, depolymerize hemicelluloses, reduce the formation of inhibitors that hinder carbohydrate hydrolysis, require low energy expenditure, and recover value-added products such as lignin.

To confirm the efficiency of the alkaline treatment, the determination of the chemical composition of hemp biomass after NaOH treatment was performed and compared to the chemical composition of the biomass before pretreatment. The results are presented in Table 3.

The analysis of the chemical composition of the hemp biomass before and after treatment showed that, in all the four varieties, the alkaline treatment resulted in a visible increase in the cellulose content (by approximately 10%) and the partial degradation of hemicelluloses (as much as 12% for the Rajan variety). The highest content of cellulose after treatment was found in the following varieties: Tygra, Białobrzeskie, and Rajan, and its level was approximately 60%. In the case of the lignin content, only for the Białobrzeskie hemp biomass, a very slight reduction was observed after the alkaline pretreatment. In the case of the remaining varieties, the tendency was contrary. Similar observations were reported by Stevulova et al. [13], who examined the chemical composition of hemp biomass before and after the pretreatment with sodium hydroxide and proved that the content of lignin after the pretreatment was 7% higher than before.

At this stage, due to better properties, availability, and higher yield, only two types of biomass of the Tygra and the Rajan varieties were selected, for which further research on bioethanol production was continued.

**Figure 1:**
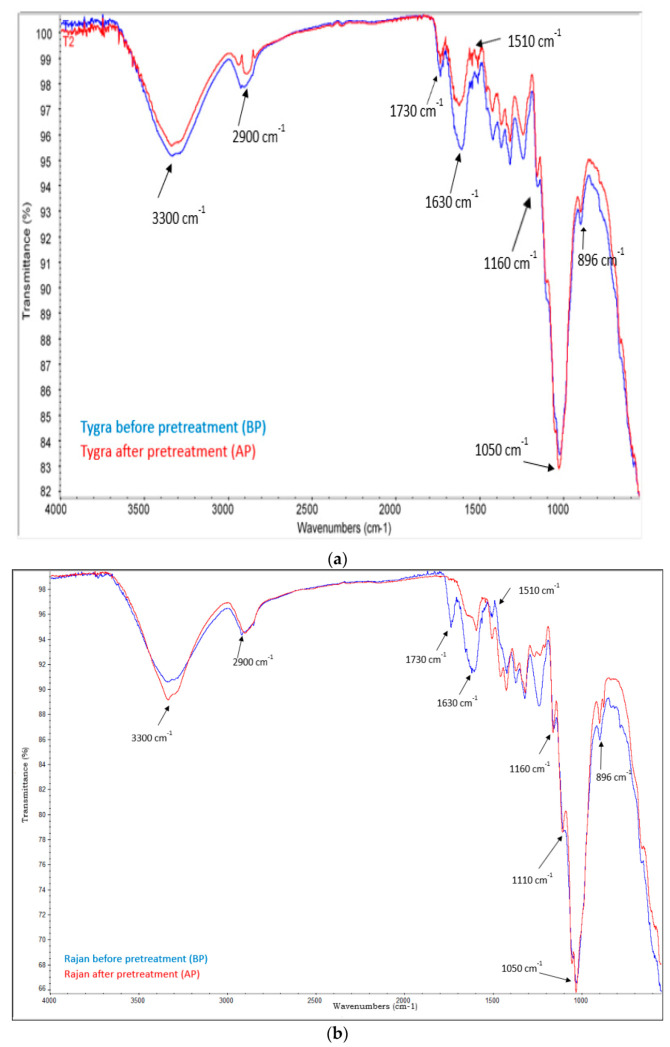
(**a**) The FTIR spectra of the Tygra biomass before and after the alkaline treatment. (**b**) The FTIR spectra of the Rajan biomass before and after the alkaline treatment.

**Figure 2:**
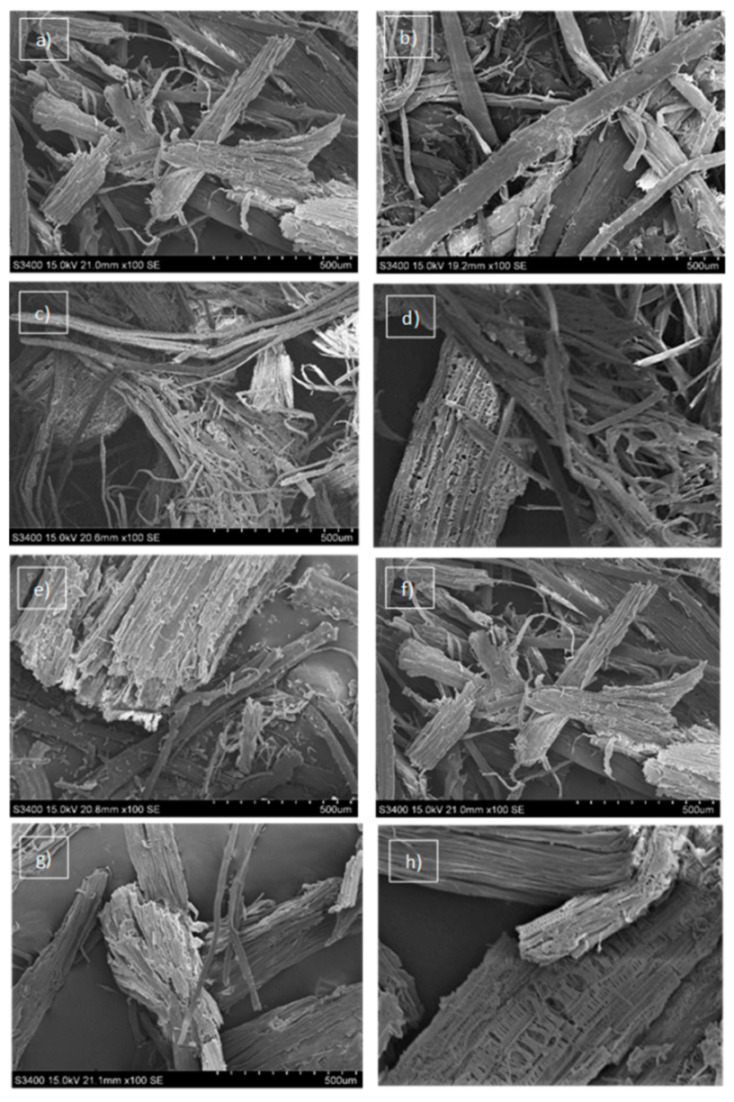
The SEM images of hemp biomass: (**a**) Tygra biomass before pretreatment, (**b**) Tygra biomass after pretreatment, (**c**) Tygra biomass after enzymatic hydrolysis, (**d**) Tygra biomass after enzymatic hydrolysis—selected fragment in high magnification, (**e**) Rajan biomass before pretreatment, (**f**) Rajan biomass after pretreatment, (**g**) Rajan biomass after enzymatic hydrolysis, (**h**) Rajan biomass after enzymatic hydrolysis—selected fragment in high magnification.

The effect of the alkaline treatment on Tygra and Rajan hemp biomass was also confirmed by Fourier transform infrared spectrometer (FTIR) shown in Figure 1a,b and by scanning electron microscopy (SEM) shown in Figure 2.

An effective method for studying the structure of biomass after alkaline treatment is FT-IR [14]. Figure 1a,b show the changes in FTIR spectra after the alkali treatment of hemp biomass between 600 cm^−1^ and 4000 cm^−1^. On the spectra of both varieties, typical vibration bands in the cellulose molecule were observed at 3300 cm^−1^, 2900 cm^−1^, and 1610 cm^−1^. The broad band in the 3600–3100 cm^−1^ region, which was due to the OH stretching vibration, gave considerable information concerning the hydrogen bonds. The peaks characteristic of hydrogen bonds from the spectra of the Rajan variety AP became a little sharper and more intense compared to the Rajan variety BP. However, in the case of the Tygra variety, this band was less intense. The 2900 cm^−1^ peak corresponding to the C–H stretching vibration in the case of the Tygra variety shifted to higher wavenumber values and slightly decreased in the intensity. These changes could have resulted from both the increased amount of cellulose after the treatment (2.2, Table 3) and the reduced cellulose crystallinity [15,16]. The band at 1610 cm^−1^ from the stretching vibrations of the O–H bonds, due to the adsorbed water in the sample, decreased, especially for the Rajan variety, which could be attributed to water loss due to drying the sample [17]. The intensities of peaks at 1160−1170 cm^−1^ (asymmetric C−O−C stretching from cellulose) and 1110−1120 cm^−1^ (C−OH skeletal vibration in cellulose) increased after pretreatment in both types of biomass. Furthermore, the intensity at 1050−1060 cm^−1^ (C−O−C pyranose ring skeletal vibration ascribed to cellulose) also increased slightly after alkali pretreatment. These changes were confirmed by the increase in the concentration of cellulose in the pretreated biomass in the chemical composition tests (2.2, Table 3) [18].

The vibration band visible at 1730 cm^−1^ (C=O stretching of acetyl groups in hemicelluloses and aldehydes in lignin) [19] was reduced in both hemp varieties after alkaline treatment, while the change was much more significant for the Rajan variety where the band almost disappeared. This occurred due to the decomposition of hemicelluloses and the solubilization of lignin during alkali pretreatment. This result correlates very well with the obtained results of the chemical composition of the biomass after pretreatment. According to these studies, the content of hemicelluloses after alkaline treatment in the Rajan variety decreased by as much as 12% (2.2, Table 3).

Moreover, the obtained FTIR spectra showed that the removal of lignin in the alkaline treatment process was problematic (band at 1510 cm^−1^). This was confirmed by the results of the chemical composition of the lignin content presented in Table 2. This problem is widely described in the literature dealing with the studies of lignocellulosic biomass by infrared spectroscopy [20,21]. The process of the degradation or fragmentation of lignin is complicated due to the presence of strong C–C bonds and other functional groups, such as aromatic groups [13].

Significant changes on the surface of the biomass were observed and presented in the SEM images taken before and after the biomass pretreatment as well as after enzymatic hydrolysis (Figure 2). In the case of both varieties of hemp biomass (Tygra and Rajan), similar changes were observed in the biomass surface, which appeared as a result of subsequent processes. However, in the case of the Rajan variety, changes were more intensive, especially after the enzymatic hydrolysis process. The untreated hemp biomass was observed to have intact, rigid, and coarse structures with smooth surface and a well-ordered fiber skeleton (Figure 2a,e). This strongly blocked access to cellulose to limit enzymatic attack [22]. As a result of pretreatment, the biomass underwent various specific structural changes. The SEM images of hemp biomass after pretreatment showed that the surface area of the biomass was partially purified (Figure 2b,f). The morphological changes that indicated damage to the structure of biomass and that increased the surface area, making it more accessible to the cellulolytic enzymes [20,23,24], were observed. The enzymatic hydrolysis of the samples subjected to the previous alkaline treatment caused further significant changes in the structure of the biomass visible in the SEM pictures (Figure 2c,d,g,h). The appearance of micropores was very characteristic here.

Undoubtedly, the opening of the hemp biomass and creating the holes all over the biomass enhanced the enzyme accessibility of the structure and facilitated biomass digestibility [25]. Moreover, it was clearly visible that enzymatic hydrolysis of the Rajan hemp biomass made the fibrous structure fragile and was more successful.

### 2.3. Evaluation of Enzyme Preparations

The enzymatic hydrolysis process is the second main step in the process of obtaining bioethanol from plant biomass. Enzymatic hydrolysis determines the amounts of simple sugars that are metabolized by yeast in the fermentation process. The breakdown of cellulose into simple sugars requires the synergistic action of different enzymes: cellulases, endoglucanases, cellobiohydrolases, and ß-glucosidases. First, the commercial enzyme preparations of various compositions were gained (Flashzyme Plus 200, ACx8000L, Celluclast 1.5L, Cellobiase, Xylanase), and then their cellulolytic and xylanolytic activities were determined. Taking into account evaluated activity of the tested enzymes and their commercial availability, Flashzyme Plus 200 and Celluclast 1.5L preparations were selected for further research (Table 4).

In order to select the enzyme complex for the SHF and the SSF processes, enzymatic tests were performed using selected enzymes and their supplementation with glucosidase and xylanase and were partially described in our previous studies [4]. For the SHF process in the case of the Tygra biomass, an enzyme complex was selected with the composition of Flashzyme Plus 200, glucosidase, and xylanase, while composition for the Rajan was Flashzyme Plus 200:Celluclast 1.5L hemp biomass in the proportion of 70:30. For the SSF process, the enzyme complex for the Tygra biomass was selected as Flashzyme Plus 200:Celluclast 1.5L (70%:30%) and xylanase and for the Rajan was Flashzyme Plus 200:Celluclast 1.5L hemp biomass in the 50:50 ratio.

### 2.4. Separate Hydrolysis and Fermentation (SHF)

To determine the optimal conditions of the enzymatic hydrolysis method as a separate process of SHF, based on the literature data and the research experience, the following parameter ranges were selected for testing with the response surface methodology (RSM): dose of the enzyme 10–30 FPU·g^−1^ of solid, temperature 50–70 °C, pH 4.2–5.4, and time 24–72 h. The RSM method is an effective optimization tool consisting of mathematical and statistical techniques and used for the process of optimization [26,27,28].

**Figure 3:**
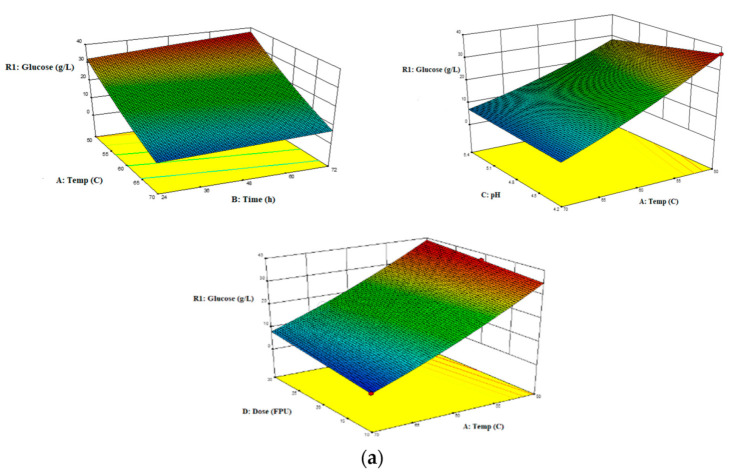

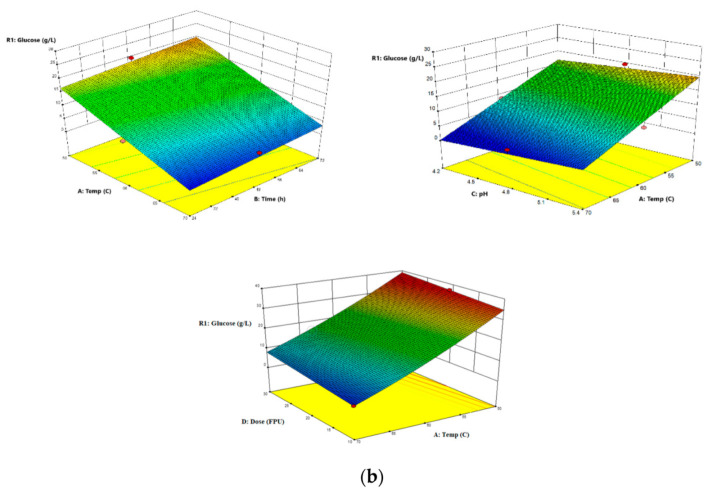
Enzymatic hydrolysis process of hemp biomass (RSM). Response surface representing the interaction effects of temperature, time, dose, and pH on glucose yield: (**a**) Tygra biomass, (**b**) Rajan biomass.

Individual enzymatic tests of the hydrolysis process were performed for Tygra and Rajan biomasses, and the evaluation criterion was the amount of released glucose. Figure 3 presents various response surfaces and interaction effects of variables (temperature, time, enzymes’ dose, and pH) on the glucose yield. The variable that had the most significant impact on the glucose content turned out to be the temperature. The lower the temperature was, the higher the glucose content was. The pH of the solution, the process time, and the enzymes’ dose had lesser effects. However, slight differences were observed in the dependence of these variables on the glucose yield for the two biomass varieties. For the Tygra biomass, it was found that, in the SHF process, the optimal conditions for enzymatic hydrolysis were obtained for the substrate concentration of 5% using the following enzymes: Flashzyme Plus 200 30 FPU·g^−1^ of solid, glucosidase 20 CBU·g^−1^ of solid, and xylanase 500 XU·g^−1^ of solid. The process parameters were: temperature 50 °C, pH 4.2, and time 48 h. These parameters provided the opportunity to obtain a maximum glucose yield, which was 36.9 ± 0.64 (g·L^−1^). In the SHF process for the Rajan biomass, optimal enzymatic hydrolysis conditions were obtained for a substrate concentration of 5% using the Flashzyme Plus 200:Celluclast 1.5L (70:30) enzyme complex with a dose of 10 FPU·g^−1^ of solid. The process parameters were: temperature 50 °C, pH 5.4, and time 72 h. The maximum glucose yield was 23.66 ± 0.16 (g·L^−1^).

Similar research was conducted by Abraham [29]; during biomass hydrolysis at 50 °C and 18 FPU·g^−1^ of solid, the highest glucose yield was obtained. Salimi and others [30] optimized the enzymatic hydrolysis of lignocellulosic biomass using the RSM method. They applied the temperature range of 45–60 °C and the pH of 4.5—6.0. They obtained the highest content of monosaccharides at 45 °C and pH 6.0. Jambo et al. [31], in turn, optimized lignocellulosic biomass using similar parameters—temperature (30–60 °C), pH (3.8–5.8), and incubation time (12–72 h)—and obtained the glucose concentration at the level 24.24 g·L^−1^.

**Figure 4:**
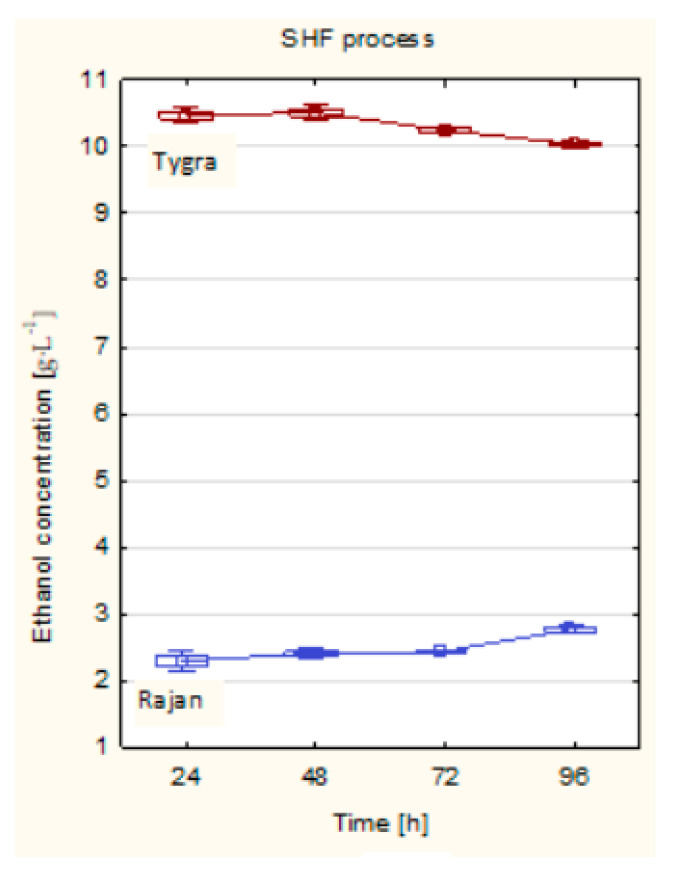
The ethanol concentration of hemp biomass in the SHF process. Optimized process conditions: Tygra biomass—substrate concentration 5%, enzymes: Flashzyme Plus 200 30 FPU·g^−1^ of solid, glucosidase 20 CBU·g^-1^ of solid and xylanase 500 XU·g^−1^ of solid, temperature 50 °C, pH 4.2, and time 48 h. Rajan biomass—substrate concentration 5%, Flashzyme Plus 200:Celluclast 1.5L (70:30) enzyme complex with a dose of 10 FPU·g^−1^ of solid, temperature 50 °C, pH 5.4, and time 72 h.

The next step in the conversion of hemp biomass to bioethanol in the SHF process was ethanol fermentation. In the SHF process for the Tygra biomass, the highest concentration of ethanol was observed at 48 hours and was 10.51 g·L^−1^. In the following hours of the process, no significant increase in ethanol concentration was observed. In turn, for the Rajan biomass, the highest ethanol concentration was noticed at 96 h, and it was only 2.76 g·L^−1^. Such a low concentration of ethanol in this case could be attributed to various reasons, which together may have had a significant negative impact on this parameter—for example, the chemical composition, which was characterized by a higher lignin content compared to the Tygra biomass. The reason could also have been the use of a low enzyme dose (10 FPU g^−1^ solid) in Rajan biomass as well as the low glucose concentration (23.66 g L^−1^), which was determined immediately after the enzymatic hydrolysis step. Additionally, after optimizing this step, a pH of 5.4 was chosen based on the glucose concentration, which may have, to some extent, inhibited yeast activity in the initial phase of fermentation. Nevertheless, it was noticed that, with each passing day, the concentration of ethanol increased slightly, which ultimately indicates that the process itself was proceeding correctly. However, for Tygra biomass, no significant increase in ethanol concentration was observed with time extension of the SHF process (Figure 4). The average yield of bioethanol for the Tygra variety was equal to 253 L∙Mg^−1^ (of hemp straw dry matter), i.e., 3.04 m^3^·ha^−1^. For the Rajan variety, the average yield of bioethanol was 69 L∙Mg^−1^ (of hemp straw dry matter), i.e., 0.80 m^3^·ha^−1^. A similar study was presented by Kusmiyati et al. [32]. In their work, the conversion of the lignocelullosic biomass to bioethanol was carried out through pretreatment, saccharification, and fermentation processes. Their results showed that the SHF process gave a higher concentration of ethanol (8.11 g·L^−1^). Fischer and others [33], in their research, dealt with lignocellulosic biomass and examined the SHF process, obtaining the ethanol concentration of 12.1 g·L^−1^.

SHF as an alternative process in an industrial bioethanol plant manifests both potential and limitations. The main advantage of SHF is the possibility to optimize the process steps separately, especially to be able to run the enzymatic hydrolysis at an optimal temperature with respect to enzymes [34]. However, most literature reports confirm that SSF is a more promising and advantageous approach with respect to SHF because of a low production cost, less processing time, less reactor volume, higher ethanol productivity, a lower requirement of enzyme, the ability to overcome enzymatic inhibition by simultaneous end-product removal, and a lower requirement for sterile conditions, as bioethanol is produced immediately with glucose conversion [35,36].

### 2.5. Simultaneous Saccharification and Fermentation (SSF)

The simultaneous hydrolysis and fermentation must be carried out under conditions that ensure the optimal synergy of enzymes and distillery yeast. To optimize the SSF process according to the RSM, the following ranges of process parameters were selected: substrate content 5%–7% *w*/*v*, the dose of Flashzyme:Celluclast 1.5L enzymes (50:50) 10–30 FPU·g^−1^ of solid using *S. cerevisiae* yeast at 37 °C, pH 4.8, and 96 h. Then, the fermentation tests were carried out using the selected parameters, and the amount of ethanol (HPLC) was determined. The optimal conditions of the SSF process for the Tygra and the Rajan hemp biomasses were selected. The highest ethanol concentration for Tygra biomass was observed at a substrate content of 5% *w*/*v* and a dose of enzyme at 30 FPU·g^−1^ of solid, and it was 6.5 g·L^−1^. In turn, for the Rajan biomass, the highest ethanol concentration equal to 7.5 g·L^−1^ was recorded for the substrate of 5% and for the enzyme 30 FPU·g^−1^ of solid. Higher substrate content above 5% *w*/*v* interfered with the effective mixing of the fermentation solution, which resulted in poorer access to biomass and thus less effective action of enzymes and yeast. It was also observed that enlarging the enzyme dose enhanced the conversion of cellulose to glucose and thus increased the concentration of ethanol. The enzyme dose of 10 FPU·g^−1^ of solid tested in the optimization process turned out to be too low to carry out efficient enzymatic hydrolysis.

**Figure 5:**
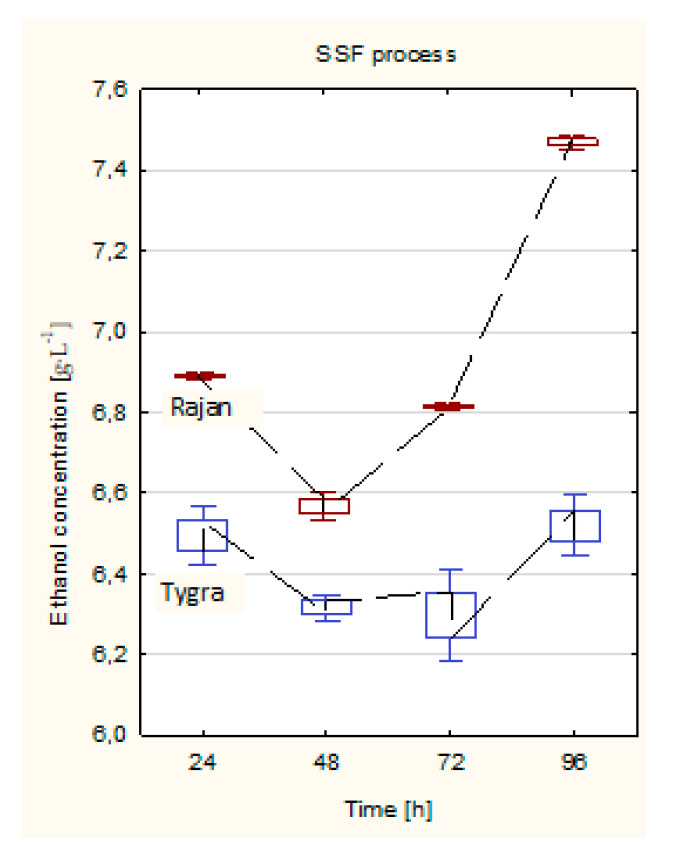
Ethanol concentration of hemp biomass in the SSF process. Optimized process conditions: Tygra and Rajan biomass-substrate concentration 5%, enzymes: Flashzyme:Celluclast 1.5L (50:50) 30 FPU·g^−1^ of solid, temperature 37 °C, pH 4.8, and time 96 h.

For the Tygra biomass, there were no changes in the concentration of ethanol with the time lapse of the process. In turn, for the Rajan biomass, after 24 h, there was observed a decrease in ethanol concentration, and after 48 h of the SSF process, there was noticed a significant increase in ethanol concentration (Figure 5). It was observed that, for the Rajan biomass, in contrast to the Tygra biomass, a higher ethanol concentration was obtained in the SSF process than in the SHF process (Figure 4 and Figure 5).

In their research, Fojas and Rosario [37] optimized the enzymatic saccharification of lignocellulosic biomass, and the SSF process was carried out with the following parameters: 3%–6% the amount of substrate, 20–25 FPU·g^−1^ of solid dose of enzyme, and temperature of 37 °C for 120 h. They achieved an ethanol content of about 9 g·L^−1^.

Research on obtaining bioethanol from hemp biomass was also carried out by Orlygsson [38]; after the SSF process, the author obtained an ethanol concentration of approximately 1 g·L^−1^.

On the basis of the average ethanol content, the hemp straw yield that could be obtained from 1 ha of hemp cultivation was specified. The highest average yield of bioethanol was estimated for the Rajan variety and was equal to 190 L∙Mg^−1^ (of hemp straw dry matter), i.e., 2.23 m^3^·ha^−1^, while the average yield of bioethanol estimated for the Tygra variety was 165 L∙Mg^−1^ (of hemp straw dry matter), i.e., 1.81 m^3^·ha^−1^.

Extensive research on industrial hemp as potential raw material for bioethanol production compared to other raw materials such as kenaf and sorghum was conducted by Das et al. [39]. According to these studies, the ethanol yield from hemp was 250 L∙Mg^−1^, which turned out to be much higher than that of kenaf. Moreover, the cost analysis allowed the researchers to conclude that industrial hemp can generate higher gross profits per hectare than other crops. In conclusion, this scientific report emphasized that hemp has the potential to be a promising crop for the production of bioethanol.

## 3. Materials and Methods

### 3.1. Bioethanol Production Process

#### 3.1.1. Hemp Biomass Preparation

The raw materials used in the study were Białobrzeskie, Tygra, Henola, and Rajan hemp (*Cannabis sativa* L.) biomasses from the Experimental Farm of the Institute of Natural Fibres and Medicinal Plants in Pętkowo. This material was subjected to preliminary crushing to the particles of size 20–40 mm and then dried in 50–55 °C for 24 h. Next, the material was disintegrated on a knife mill (Retsch SM-200, Germany) with the sieves of the mesh size of 2–4 mm. An enzymatic test for the crushed fractions was performed using the Celluclast 1.5L enzyme preparation, and the content of reducing sugars was determined by the Miller’s method with 3,5-dinitrosalicylic acid (DNS) [40].

#### 3.1.2. Alkaline Pretreatment

The evaluation of pretreatment conditions for hemp biomass was carried out at 5 h treatment with 1.5%–3% sodium hydroxide in 90 °C. NaOH:biomass weight ratio was 10:1. After the alkaline pretreatment was carried out, the biomass solution was filtered on a Büchner funnel, then washed with distilled water until neutralized and dried in a laboratory dryer at 50 °C for 24 h. The alkali effect on the biomass was evaluated in the enzymatic test, and content of the released reducing sugars was determined by the Miller’s method. This test was performed with the use of Celluclast 1.5L (Novozymes, Bagsværd, Denmark) enzymatic preparation at the dose of 10 FPU·g^−1^ of solid. The raw material was incubated at 55 °C in 0.05 M citrate buffer of pH 4.8 for 24 h. Then, after the enzymatic test, the supernatant was diluted, a DNS reagent was added, and the mixture was incubated in a boiling water bath for 10 min. After cooling to room temperature, the absorbance of the supernatant was measured at 530 nm on UV–VIS Spectrophotometer V-630 (Jasco, Pfungstadt, Germany).

#### 3.1.3. Enzyme Complex

The enzymes cellulolytic activity was determined according to NREL LAP Measurement of Cellulase Activities. In turn, enzymes xylanolytic activity was determined according to the Osaka University procedure (with changes) [41].

In order to select the enzyme complex for SHF and SSF processes, tests were performed using selected enzymes (Flashzyme Plus 200:Celluclast 1.5L) and their supplementation with glucosidase 20 CBU·g^−1^ of solid and xylanase 500 XU·g^−1^ of solid (Sigma-Aldrich, Darmstadt, Germany ). Enzymatic tests were carried out for 5% of biomass with the enzyme in the amount of 10 FPU·g^−1^ of solid, at a pH of 4.8, and during 24 h at 55 °C for the SHF process and at 38 °C for the SSF process. The selection criterion was the content of reducing sugars determined by the Miller’s method.

#### 3.1.4. Separate Hydrolysis and Fermentation (SHF)

The optimization of the enzymatic hydrolysis of hemp biomass in the SHF process was carried out according to the response surface methodology (RSM) using the parameters: biomass content 5%–7% *w*/*v*, temperature 50–70 °C, time 24–72 h, pH 4.2–5.4, dose of enzyme 10–30 FPU·g^−1^ of solid. Then, tests of the hemp biomass hydrolysis process were performed, and the evaluation criterion was the amount of released glucose.

In the next stage, the obtained hydrolyzate was subjected to the ethanol fermentation process carried out in bioreactor Biostat B Plus (Sartorius, Göttingen, Germany) in 2 L vessel equipped with pH, temperature, stirring, and foaming controls. The temperature was maintained at 37 °C and stirring at 900 rpm, while pH was controlled at 4.2 for Tygra and 5.4 for Rajan by adding 1 M NaOH or 1 M HCl. Non-hydrated freeze-dried distillery yeast *S. cerevisiae* (Ethanol Red, Lesaffre, France) at a dose of 1 g·L^−1^ was used in the process, which corresponded to cell concentration after inoculation of about 1 × 10^7^ cfu/mL. Inoculum grew for 24 h at 30 °C. After inoculation, a 96 h fermentation was carried out, and samples were taken every 24 h. All experiments were performed in triplicate.

#### 3.1.5. Simultaneous Saccharification and Fermentation (SSF)

To optimize the SSF process according to the RSM, the ranges of process parameters were selected: substrate content 5%–7% *w*/*v*, dose of (Flashzyme:Celluclast 1.5L) enzymes 10–30 FPU·g^−1^ of solid. The SSF process was carried out in bioreactor Biostat B Plus (Sartorius, Göttingen, Germany) in 2 L vessel equipped with pH, temperature, stirring, and foaming controls. The temperature was maintained at 37 °C and stirring at 900 rpm, while pH was controlled at 4.8 by adding 1 M NaOH or 1 M HCl. In the fermentation process, non-hydrated freeze-dried distillery yeast *S. cerevisiae* (Ethanol Red, Lesaffre, France) at a dose of 1 g·L^−1^ was used, which corresponded to cell concentration after inoculation of about 1 × 10^7^ cfu/mL. The duration of ethanol fermentation was 96 h. All experiments were performed in triplicate.

### 3.2. Analytical Methods

The chemical composition of hemp biomass before and after pretreatment was determined, i.e., cellulose according to TAPPI T17 m-55 [42], hemicelluloses as the difference of holocellulose and cellulose according to TAPPI T9 m-54 [43], and lignin according to TAPPI T13 m-54 [44].

In order to provide a more complete picture of the molecular structure of hemp biomass before and after the chemical pretreatment, the analysis of FTIR spectroscopy was performed using a Fourier Transform Infrared Spectrometer ISS 66v/S (Bruker, Bremen, Germany) at infrared wavenumbers of 400–4000 cm^−1^ [13].

The physical morphologies of hemp biomass before and after the chemical treatment and after enzymatic hydrolysis were performed using Scanning Electron Microscope S-3400N (Hitachi, Japan) in high vacuum conditions. The samples were covered with gold dust.

The contents of glucose and ethanol were determined by high performance liquid chromatography on Elite LaChrom (Hitachi, Tokio, Japan) using an RI L-2490 detector, Rezex ROA 300x7.80 mm column (Phenomenex, Torrance, CA, USA), as the mobile phase used 0.005 N H_2_SO_4_ at a flow rate of 0.6 mL/min at 40 °C.

### 3.3. Calculations

The ethanol yield from 100 g of raw material Y_s_ (g/100 g of raw material) was calculated according to the Equation (1) [45]:

(1) $$Y_{s}=\frac{\;Et\times 100}{M}$$

where: *Et*—amount of ethanol in 1000 mL of tested sample (g); *M*—mass of material weighed in 1000 mL fermentation sample (g).

Then, based on the ethanol yield from 100 g of raw material, the amount of ethanol in L per ton of straw dry matter (L∙Mg^−1^) was calculated, and on the basis of straw yield, the ethanol yield per hectare (m^3^∙ha^−1^) was determined.

### 3.4. Statistical Analysis

The experiments of ethanol fermentation were carried out in triplicates. Standard deviations were calculated using the analysis of variance ANOVA, Statistica 13.0 software (*p* < 0.05).

## 4. Conclusions

To sum up, the Tygra and the Rajan varieties of hemp were selected, which proved to be proper sources of second generation bioethanol as alternatives to petroleum-oil based fossil fuels. Pretreatment, enzymatic hydrolysis, and ethanol fermentation were optimized. Alkaline pretreatment caused an increase in cellulose content and partial degradation of hemicelluloses. Enzymatic hydrolysis allowed us to achieve glucose yield at the level up to 36.9 g∙L^−1^. For the Tygra biomass in the SHF process, the ethanol concentration was 10.5 g∙L^−1^ (3.04 m^3^·ha^−1^), and for the Rajan biomass in the SSF process, the ethanol concentration was 7.5 g∙L^−1^ (2.23 m^3^·ha^−1^).

In the future, it will be important to conduct research on the mixtures of different varieties of hemp biomass in order to determine their potential for the production of lignocellulosic ethanol, which seems important in practical application, because the industrial production of biofuels occurs most often in large refineries which process the biomass of different varieties and species of plants.

## Figures and Tables

**Table 1:** The amount of reducing sugars (mg·g^−1^) released after an enzymatic test depending on the fraction size.

Hemp Biomass	4 mm	2 mm
Białobrzeskie	65.1 ± 0.06	68.5 ± 0.26
Tygra	73.3 ± 0.14	76.3 ± 0.17
Rajan	51.3 ± 0.32	57.8 ± 0.04
Henola	50.4 ± 0.07	54.8 ± 0.16

**Table 2:** The amount of reducing sugars (mg·g^−1^) released after an enzymatic test depending on the NaOH concentration.

Hemp Biomass	1.5%	2%	3%
Białobrzeskie	163 ± 0.78	178 ± 1.37	173 ± 0.23
Tygra	183 ± 1.43	206 ± 0.87	203 ± 1.70
Rajan	190 ± 2.54	180 ± 0.68	176 ± 0.55
Henola	159 ± 1.86	166 ± 1.15	147 ± 1.33

**Table 3:** The chemical composition of hemp biomass (percentage of dry matter); BP: before pretreatment; AP: after pretreatment.

Variety	Samples	Cellulose (%)	Hemicelluloses (%)	Lignin (%)
Białobrzeskie	BP	50.10 ± 0.18	32.10 ± 0.22	15.40 ± 0.03
	AP	61.46 ± 0.37	21.59 ± 0.06	15.12 ± 0.16
Tygra	BP	50.82 ± 0.12	27.79 ± 0.33	14.68 ± 0.46
	AP	62.70 ± 0.09	20.16 ± 0.16	15.12 ± 0.22
Henola	BP	46.82 ± 0.04	29.94 ± 0.45	15.48 ± 0.17
	AP	57.62 ± 0.08	20.33 ± 0.22	17.80 ± 0.06
Rajan	BP	48.69 ± 0.39	31.43 ± 0.04	16.72 ± 0.08
	AP	59.30 ± 0.33	19.91 ± 0.25	18.40 ± 0.18

**Table 4:** The determination of cellulolytic and xylanolytic activities of commercial enzyme preparations (FPU·mL^−1^).

Enzyme Preparations	Composition	Cellulolytic Activity	Xylanolytic Activity
Flashzyme Plus 200	endoglucanase, cellobiohydrolase, cellobiose, xylanase, mannase	90	2430
ACx3000L	endo-1,4-β-D-glucanase	63	487
Celluclast 1.5L	cellulase, xylanase	62	278
Accellerase 1500	exoglucanase, endoglucanase, cellobiose, β-glucosidase, hemicellulase	53	616
Alternafuel CMAX	cellulase, β-glucosidase, hemicellulase, arabinose	2.78	110
ACx8000L	cellulase	8	190
Cellobiase	glucosidase	0.18	325
Xylanase	xylanase	0.03	746

## Data Availability

Data is contained within the article.
